# How Learning Culture Influences the Survivability of an Online Feedback Tool

**DOI:** 10.5334/pme.2166

**Published:** 2026-03-23

**Authors:** Su Ann Khoo, Yang Yann Foo, Lorelei Lingard, Arif Tyebally

**Affiliations:** 1 Department of Emergency Medicine (Pediatrics), KK Women’s and Children’s Hospital, Singapore; 2 Office of Education, Duke-NUS Medical School, Singapore; 3 Schulich School of Medicine & Dentistry, Western University, Canada

## Abstract

**Introduction:**

Many online tools and innovations exist to support feedback, but their survivability has not been well-studied. This study explores the influence of learning culture on the adoption, utilization and long-term impact of an online written feedback tool among faculty. The study aims to inform strategies for effective implementation of such tools and guide faculty development within the clinical learning environment.

**Methods:**

Utilizing constructivist grounded theory, we conducted semi-structured interviews among ten clinical faculty with supervisory roles and experience in giving medical trainees feedback via an online feedback tool. Using an iterative analytic approach, we identified key propositions to derive an in-depth understanding between learning culture (LC) and the “survivability” of the online feedback tool.

**Results:**

Several socio-cultural factors within the LC have affected the survivability of the feedback tool beyond its development and implementation. Firstly, the lack of a support system exacerbated the burden of clinical teachers. Next, the misapprehension about written feedback and low psychological safety undermined the tool adoption. Lastly, the lack of appreciation towards the importance of a strong feedback loop further reduced the tool’s survivability.

**Conclusion:**

The development and implementation of online tools in health professions education should take into consideration factors influencing the survivability of the tool. The exploration from a socio-cultural perspective has provided insights to ensure that future online feedback tools and interventions actually survive the test of time and the complexities embedded in the clinical learning environment.

## Introduction

Feedback is known to powerfully influence medical trainees’ performance and improvement [1]. However, trainees have persistently reported that the quality and quantity of feedback from faculty is vague and inadequate [2,3,4]. Feedback challenges in the Emergency Medicine (EM) setting are arguably especially pronounced. EM trainees rely on feedback from multiple clinical faculty during their shifts to guide their performance and development, unlike their counterparts in procedure-based specialties who experience more consistent, longitudinal supervision by their mentors [5]. EM supervisors may have brief, fleeting encounters with trainees, making it challenging to develop a comprehensive understanding of their performance. As a result, they may struggle to engage trainees in meaningful discussions and reflection during scheduled feedback sessions, due to the lack of consolidated and longitudinal information on which to provide feedback [5,6,7].

Technological innovations are a popular response to augment feedback interactions. Online tools to gather multi-sourced feedback in the learning environment have been created for both medical students and teachers [8,9]. However, studies that seek to understand factors that influence the survivability of technological feedback innovations have been scarce [10,11,12]. Despite the vast potential of digital innovation and online tools and their implementation in health professions education, there is little understanding of how to promote the survivability and impact of the tools’ implementation in the clinical learning environment. The term ‘survivability’ is favoured over ‘sustainability’ for this study. ‘Survivability’ of a tool is its ability not just to sustain but to thrive despite ongoing complex and dynamic challenges within its environment. By contrast, a tool can actually be sustained even with patchy usage.

The survivability of any educational tool or innovation can be challenging in a complex clinical learning environment. In the case of our online written feedback tool developed to provide feedback to trainees rotating in the Emergency Department (ED), we encountered issues related to faculty motivation and sustained adoption of the tool. Through discussion with faculty, we realized that the uptake impediments had less to do with technical issues but more to do with their preconceived notions about written feedback that were socio-cultural in nature (see more under Methods section). Several studies have highlighted the valuable insights that socio-cultural perspectives could provide [13,14,15]. One potentially useful concept may be that of learning culture or LC (Watling, 2015; Watling et al, 2013b). This study aims to understand how LC influenced the survivability of an online written feedback tool based on its perceived usefulness.

## Methods

Constructivist grounded theory (CGT) was utilized to explore how LC influences the survivability of a written feedback-related tool among faculty in the pediatric emergency department (the clinical learning environment) of KK Women’s and Children’s Hospital in Singapore. CGT enables exploration of social processes and interactions [16,17], and enables us to pay attention to social origins of our participants’ values, beliefs and attitudes that influence their feedback practices [18,19].

### Setting and innovation

The study took place at the ED of a tertiary paediatric hospital which attends to 170,000 patients a year and where 40 to 50 trainees rotate through every six months for training. SA and AT developed an online written feedback tool (refer to Appendix 2) to improve the quality and quantity of written feedback provided by faculty to trainees in the ED. “Faculty” refers to the senior doctors in the ED who play the roles of clinicians, educators and supervisors. Each senior doctor has between 10 to 30 years of supervisory experience.

In our ED setting, the online written feedback tool was made accessible to faculty who served as either “clinical supervisor” or “shift supervisor”. The term “clinical supervisors” refers to faculty who are assigned to be the overall assessor and supervisor of a trainee throughout the posting and may not necessarily work with the trainee during every shift due to the nature of shifts in the ED. Clinical supervisors were meant to be the recipients of the written feedback so that they can provide specific and actionable guidance to their trainees for improvement. The term “shift supervisors” refers to faculty who work with trainees during shiftwork. As shift supervisors had opportunities to observe trainees’ interactions with patients and other healthcare professionals, their written feedback was thought to hold tremendous value for improvement.

The online written feedback tool was developed to address a significant problem: the trainees’ assigned clinical supervisors did not always have sufficient contact with the residents throughout their posting to have a holistic view of their performance because of shift work. This meant that at scheduled meetings, clinical supervisors often could not provide sufficient feedback based on their personal observations to help the supervisees improve. SA and AT thus designed the tool to enable the longitudinal collection of written feedback from multiple faculty members who worked with trainees in the ED. The tool was designed to encourage faculty to provide feedback across all 6 of the Accreditation Council for Graduate Medical Education-International (ACGME-I) core competencies. The tool could be conveniently accessed by faculty via their smart phones to submit real time written feedback related to clinical consultations, procedural-skill observations, simulation sessions, didactic teaching, presentations or research-related work. The collection of written feedback from various faculty was intended to provide a more holistic picture of trainees’ performance based on observations. Faculty were also taught how to use the R2C2 framework (R-Relationship, R-Reactions, C- Content, C-Coaching) [20] during supervisor-supervisee meetings. R2C2 allowed faculty to have more meaningful discussions and reflections during those scheduled meetings with their trainees during their posting. As teaching faculty with prominent roles in medical education, SA and AT collaborated with faculty to innovate feedback practices to improve the quantity and quality of feedback for trainees.

#### Online Feedback Tool: Development and Learnings Guiding its Evolution

**Diagram 1:**
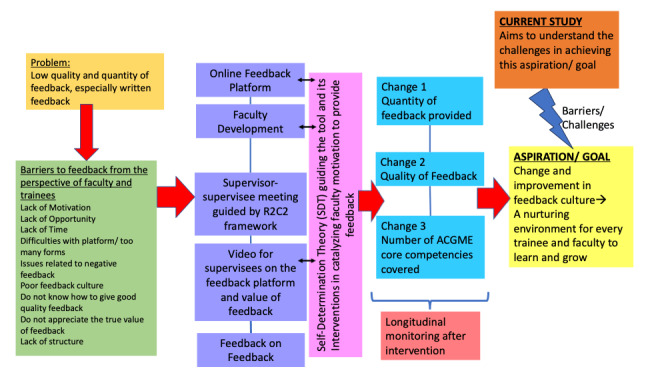
Problem-listing, needs assessment, followed by multi-pronged interventions guided by SDT and DIT to ensure the adoption and utilization of the online feedback tool.

This segment reflects the critical learnings in the journey of developing and implementing the tool. The design and implementation of our online written feedback tool were guided by self-determination theory (SDT) [21] and diffusion of innovation theory (DIT) [22]. SDT explains motivation by emphasizing an individual’s inner resources, fostering sustained, self-regulated behaviours [23] and informing faculty development initiatives [24,25]. DIT is a framework that explains how new ideas, technologies and practices spread through a social system over time and plays a vital role in understanding change in healthcare, education, organisational and implementation contexts [22]. However, despite careful considerations in the design, delivery, and implementation, as well as multi-pronged faculty interventions (illustrated in Diagram 1), the tool faced significant challenges. Despite receiving strong leadership support from the head of the department, there were variable levels of adoption of the tool among faculty. There were difficulties in motivating faculty to continue providing feedback via the tool. Although we collected and disseminated positive resident feedback highlighting the quality of faculty-resident interactions facilitated by the tool and residents’ appreciation for faculty engagement with the feedback process, achieving sustained and consistent faculty adoption of the tool proved difficult. Faculty were also overwhelmed with clinical duties and felt that the tool was a duplication, if not an additional burden, to the existing forms they already had to complete as clinical supervisors. Despite faculty development and regular meetings to engage the faculty, there was only transient improvement in their motivation and provision of feedback for trainees among faculty. The challenges of adoption and sustained use of our online feedback tool highlighted the importance of designing tools with survivability in mind, so that the tools can actually withstand institutional changes over time [14]. This tool was launched and used for nearly two years and about 80% of our faculty utilised the tool during this period. Over time, the percentage of faculty using the tool and the number of feedback submissions by faculty declined. The initial aspiration (yellow box in Diagram 1) did not materialise despite all efforts and interventions. The team was committed to re-examine the factors affecting the survivability of the feedback tool and the challenges to achieving our aspiration. We aimed to look beyond individual motivation to larger contextual perspectives within the learning culture that would inform the tool’s long-term survivability.

#### Learning culture (LC)

LC refers to the culture where learning happens [26,27]. While “learning” is central in LC, learning and feedback are inseparable in medical education [28]. LC may be broadly applied but can also be contextualised to the shared beliefs, values, attitudes and practices that shape teaching and learning within a profession [26]. A problem examined from the angle of LC provides an added sociocultural lens of “why” in understanding problems among faculty or learners. Using the concept of LC, researchers may explain why some strategies are prioritised’ over others, why some experiences are perceived to be more credible, and why certain approaches flourish while others are diminished. The earlier description of events in the manuscript reflected the amount of work done by SA and AT to prepare faculty to optimally use the feedback tool. When it did not turn out as planned, the informal conversations that SA and AT had with faculty revealed that the visible problems were really just the tip of the iceberg. A lot of reservations and resistance held by faculty were not related to technical issues such as how to use the online written feedback. Instead, what seemed to be holding back the faculty were their reservations about written feedback due to institutional practices and personal experiences.

Given the sociocultural nature of the faculty’s reservations and conceptions regarding written feedback, we used LC as a lens to examine how individuals’ and groups’ choices, practice, values, beliefs and attitudes shape faculty approaches to written feedback and the tool. A topic guide was designed for interviews to elicit faculty’s beliefs, values and attitudes held towards feedback, particularly written feedback. After the first two interviews, preliminary analysis was performed, and the team realised that LC could powerfully explain what was happening on the ground. The topic guide was iteratively modified accordingly.

#### Reflexivity

SA and AT, both ED physicians, developed the online written feedback tool and, therefore, were inclined to believe in its success. Hence, FYY, a constructivist qualitative researcher, was brought in to provide an independent perspective, having had no prior involvement with the tool. LL has explored the role of learning culture in previous studies of clinical training and feedback, as well as being an expert in constructivist grounded theory methodology.

### Recruitment and participants

Twenty-two clinical faculty from the pediatric ED at KK Women’s and Children’s Hospital (KKH), Singapore, volunteered to participate following an email invitation from the department’s administrative team. Ten faculty were interviewed based on their background and patterns of use of the online feedback tool, These faculty members worked with trainees in various roles and demonstrated varying levels of tool adoption and usage – as posting supervisors during ED rotations and/or as clinical supervisors during routine clinical shifts. The sequence of interviewing was as follows: the initial three participants were ‘frequent users’; the subsequent two participants were infrequent users who became non-users; the next two participants were initial adopters who became non-users; and the last three participants were a combination of initial non-users or infrequent users who became late adopters of the tool. We adopted theoretical sampling, a key feature in CGT, whereby participants were selected strategically to further explore propositions and gather contrasting perspectives [16,29]. Table 1 shows the definitions of the terms used in defining the frequency and length of the tool’s usage among faculty. Table 2 illustrates the profiles of the participants and their involvement in the various phases of the interviews.

**Table 1:** Definition for terms used in categorizing faculty’s usage of the feedback tool.


TERM	DEFINITION

Frequent user	Used the tool to provide feedback 2 to 3 times per week (at least 15 to 20 feedback entries per month) with sustained usage for at least 6 months

Infrequent user	Used the tool less than 2 times per week (fewer than 15 feedback entries per month), with some months having fewer than 5 feedback entries. Total tool usage was less than 6 months.

Initial adopter	Used the tool to provide feedback for the first 3 months, then stopped

Late adopter	Did not use the feedback tool initially; adopted the tool to provide feedback after 6 months

Non-user	Did not use the tool to provide feedback at all


**Table 2:** Summarized profiles of the participants and the involvement in the various phases of the interviews.


PHASE OF INTERVIEW	NUMBER OF PARTICIPANTS	POSITION	YEARS OF EXPERIENCE	FREQUENCY OF FEEDBACK PLATFORM USAGE

Initial phase	3	2 consultants, 1 senior consultant	12 to 30 years	Frequent users

Mid Phase 1	2	1 Associate Consultant, 1 Senior Consultant	10 to 20 years	Infrequent users turned non-users

Mid phase 2	2	1 Associate Consultant, 1 Senior Consultant	10 to 25 years	Initial adopters turned non-users

Final Phase	3	1 Associate Consultant, 1 Senior Consultant	10 to 30 years	Combination of initial non-users or infrequent users, turned late adopters


### Data Collection

One-on-one semi-structured interviews, guided by a topic guide (Appendix 1), were conducted via Zoom with the participants and lasted 45–60 minutes. Written informed consent was obtained. There were two interviewers, SA and AT. The interviewers strategically planned and arranged themselves to interview specific participants to improve rapport-building, build trust and minimize power differentials among participants to elicit in-depth narratives. This approach has been extremely valuable in qualitative studies [17]. The audio-recorded interviews were transcribed by an independent party to ensure anonymity, accuracy, and standardization for ease of coding and analysis. Data collection occurred simultaneously with data coding. The experiences, incidents, and perspectives of our participants helped identify concepts that shaped the next selection of participants for the interviews. The first three participants (as frequent users of the tool) provided insights into why they were motivated to use the tool longitudinally. The next group of participants, initial adopters who then became non-users, helped us explore the reasons for ‘drop-out’ and disengagement with the tool despite their initial confidence in and adoption of the tool. The final group of participants, initial non-users who later decided to adopt the tool, helped us explore why the tool did not initially appeal to them despite their being strong believers of feedback, and what subsequently changed their beliefs and decisions to adopt the tool. All these strategies fit the iterative nature of CGT to explore discrepancies as well as deepen propositions [16,30].

### Analysis

The transcripts of the interviews were analysed independently by SA, AT and YY, using manual line-by-line coding as well as NVivo software (QSR International Pty Ltd, Melbourne, Australia, 2018). A combined manual-software analysis provided opportunities for researchers to explore their data visually and strategically to foster creative thinking and stimulate the construction of insights that enhance the analytic process [31]. Researchers wrote memos to enhance reflexivity [19] and held regular meetings for constant comparison of their analysis and tweaking of the interview guide which informed the choice of subsequent sampling. LL was consulted regularly throughout the study and provided guidance on the development of the research question, expansion and deep exploration of propositions, as well as refining the interview guide to support theoretical sampling.

After 10 interviews, we determined that we had reached theoretical sufficiency in terms of being able to robustly describe how learning culture influenced the survivability of our online written feedback tool based on its perceived usefulness [32,33].

### Ethics

This study (EING1909) was approved by the SingHealth Centralised Institutional Review Board (CIRB number 2024/2049).

## Results

We conducted 10 semi-structured interviews between 1 August 2023 and 31 March 2024. The participants were ED faculty with supervisory roles as illustrated in Table 2. They were either clinical supervisors, shift supervisors or assumed both roles concurrently. They were all involved as feedback tool-users and participated in faculty feedback training sessions conducted by SA and AT.

The results of this study are presented through four propositions, which, together, afford a robust explanation of how LC influences survivability of the feedback tool in the clinical learning environment.

### The lack of support systems exacerbated the tension among faculty as clinical teachers

In healthcare education, faculty were often expected to adopt dual roles as clinicians and teachers. Yet, the LC of the clinical learning environment privileged service delivery, sidelining faculty’s need to be better clinical teachers. This invariably left them feeling that “a lot of feedback and teaching was by the way” and became “mostly accidental rather than systematic.” (P1)

Participants were fully aware of the importance and utility of the feedback tool. However, the heavy clinical workload constrained the full utilisation of the tool and this was one of the factors affecting its survivability:

“Often we are rushing [in a clinical shift] rushing to document; to have face to face conversation with the medical officers… because of the other clinical requirements or exigencies, using innovative tools might not be as regular as I would like to.” (P4)

This tension of having to balance patient care yet shouldering the responsibility of teaching and guiding new trainees was not new. However, with the lack of support systems and further beliefs illustrated below, it became a lingering shadow that influenced the tool’s survivability.

### Misapprehensions about written feedback manifest as misgivings towards the tool

Faculty’s incomplete understanding of the nature and purpose of written feedback (WF) had direct implications on the tool usage. Multiple misapprehensions emerged, each contributing to the perception that written documentation of feedback was unnecessary, duplicative, or reserved only for serious situations rather than serving its intended role in longitudinal trainee development.

Many participants expressed the view that written feedback was redundant when they had already provided verbal feedback. Faculty questioned the utility of documenting feedback that trainees had already received verbally, assuming that the spoken word was sufficient for learning:

“I think whatever I put down in the tool, I would have already given it face-to-face…I think verbal is good enough.” (P3)

The perception of redundancy extended to beliefs about the purpose and audience of written feedback. Some faculty felt that written feedback served administrative rather than developmental purposes, and assumed that trainees would remember verbal feedback without written reinforcement. Hence, faculty could not appreciate the value of feedback being documented again in the tool:

“To document it downright is purely just for the sake of the supervisor meeting, to repeat the thing again, right. I feel it’s not necessary because if I’ve already tell the person then I don’t see the need three months down the road or six months down the road, right for another person to read my feedback and then go and repeat the same thing based on my feedback.” (P10)

The misapprehension also manifested in selective documentation practices based on perceived severity thresholds. Some faculty believed that only serious or extreme feedback needed to be documented, overlooking the value of capturing routine formative observations or encounters for development tracking. This threshold mentality paradoxically created hesitancy around negative feedback due to its perceived permanence and weight and thus, influenced their decision to utilize the tool in documenting their feedback:

“…to give you a negative feedback in written words, it needs to be, it needs to be serious enough, I feel.” (P2)

Underlying these misapprehensions was a strong belief in the superiority of verbal feedback for learning. Faculty valued verbal feedback for its ‘timeliness’ and ‘effectiveness’ and contextual immediacy, not recognizing that WF could serve a complementary longitudinal function rather than a competing one:

“…the verbal [feedback], may be more effective and actually can bring across the message better and it’s timely…the person who’s receiving the feedback knows exactly why and the what and the how.” (P10)

Faculty often perceived WF as redundant since they had already provided verbal feedback. They failed to recognize that both forms of feedback served distinct and complementary purposes in a feedback system. This fundamental misunderstanding shaped their engagement with the tool, as many believed verbal feedback alone was sufficient for trainee development.

### Low psychological safety undermined trust in the tool’s intent

The department’s hierarchical and judgmental LC led to low psychological safety. Bedside tutorials, didactic teachings as well as morbidity and mortality rounds were often frowned upon as ‘clown shows’, whereby feedback delivery was perceived as a form of humiliation:

“There is this hierarchy thing … the resident might have certain questions but do not dare to question for the fear of feeling [perceived] stupid … in the past when we need to go and go for trainee or resident’s tutorials in the past with feedback, these kinds of tutorials are called clown shows… that is quite stigmatizing.” (P1)

When the LC was marked by low psychological safety, faculty interpreted feedback processes, especially those involving documentation, as potentially risky or threatening. This perception of threat undermined trust in the tool itself, reducing its acceptance, adoption, and survivability.

The healthcare system stored documentation as a permanent record. Thus, feedback captured in writing was also deemed to be a permanent ‘trace’ in the records that could adversely influence the trajectory of a trainee’s development:

“Definitely there’s also repercussion [if you document it]. If I think that is something that can be improved on…I probably wouldn’t want to put it down in writing.” (P7)

Participants shared the lack of trust with regard to who actually had access to WF. The tool became an extension of the LC that surrounded it. In an environment lacking safety, trust in the tool’s neutrality was undermined. Multiple stakeholders in the existing system were perceived to always have access to the feedback:

“I personally don’t think that it should be something written and other people get to see it. Unless it’s something like an RMS [Risk Management System]…the thing is that it may not be just a supervisor because it also goes through the whole admin team because things like complaint letter right is also through the admin right. So in those cases [feedback via the tool]…I guess the admin will be involved as well in that sense.” (P10)

Faculty shared how they felt feedback recorded in the tool then actually “passed through layers of interpretation” (P3) by supervisors and the administrative team, risking distortion of the original intent. Faculty found it hard to fully trust the tool to deliver its intended purpose of developmental feedback within the learning environment. Even when reassurances were given about the developmental intent of the feedback tool, the fear of misinterpretation, emotional exposure, or reputational consequences lingered:

“I would ask [myself] …Do I really need to document this? I could have been emotional typing this feedback and it could be taken out of context, like feedback being critiqued. What if he or she thinks how come this person goes behind and write the feedback and I [the learner] hear about it two months late right. Those are my biggest struggles…written [feedback] just carries so much weight. ” (P5).

In a LC with low psychological safety, the lack of trust inadvertently extended to the tool mediating feedback exchanges. A feedback tool that carried documented feedback was deemed to risk permanence, misinterpretation, or escalation. Despite repeated assurances, faculty appraised the feedback tool as a conduit through which judgment could flow, rather than as a developmental aid. This appraisal, rooted in fear, constrained engagement and inhibited the tool’s survivability.

### Strong feedback loops were essential for tools to survive

As with many healthcare settings, the LC was made up of opportunistic, episodic encounters between faculty and trainee. From an assessment and feedback perspective, this was suboptimal as opportunities for longitudinal observation and assessment from one supervisor would not be possible given the nature of rotations and shift work. The fleeting nature of these relationships weakened faculty’s ability to close the feedback loop, where they could see whether their feedback had been accepted and used by trainees. Given the circumstances, participants described feedback as “like a kind of recovery or repair process” that helped the trainees improve, yet noted that “we never know [the] long term [impact] because many times it really doesn’t come back to us.” (P2)

Without strong feedback loops that demonstrated how faculty’s written observations contributed to trainee development, many struggled to justify the effort required to use the tool consistently. Faculty expressed frustration at not knowing if their efforts made any difference and what the ‘downstream impact’ was:

“…I would like to know the change in [their] behaviour…the change in [their] practice…that it [the feedback] made a difference.” (P6)

The feedback loops were also crucial to many faculty as they were seen as an important channel to receive feedback from trainees for their own reflection and improvement. The opportunities for feedback on feedback were unfortunately not encountered frequently in the current feedback system:

“I think it’s important [to hear back] because sometimes we have our own jaundiced views or perhaps a limited vision because we don’t see the overall myriads of experience [of giving and receiving feedback]…So, I think feedback to us is also important and critical…if there is time ..feedback to the supervisor [by trainees] as well…that gives the overall holistic feedback experience.” (P9)

The lack of closure meant that faculty invested time and effort into using the tool to provide WF without any confirmation that it was read, understood or applied by trainees. Patient-care responsibilities rendered time-starved faculty further inability to develop meaningful relationships with trainees they met opportunistically in the ED. The episodic nature of encounters made faculty question the appropriateness of their efforts:

“It might look unfair in the trainees’ eyes during that short posting they spent with us…they would think: 95% of the time I’m doing a good job, then 5% for whatever reason, you documented a negative thing [feedback] and that carries implication to the fleeting relationship” (P7)

Beyond knowing whether feedback led to change, faculty needed signals that their feedback was valued and sought after by trainees. When trainees actively sought feedback, it was perceived that they valued it and faculty would be more motivated to use the tool:

“I don’t actually have the juniors reminding me to do it. To me, because somebody would like to have it and seek the feedback, then it becomes high on the priority for us to do it.” (P4)

This distinction was critical – participants valued learner-initiated feedback requests, rather than program mandates to provide feedback in a paternalistic manner. Trainees actively seeking feedback created a feedback loop that reinforced faculty effort by demonstrating immediate appreciation and purpose, reminding faculty that the time and effort to give feedback to a trainee was crucial as “for us [as a faculty or supervisor], it [this reminder to give feedback] becomes immediately to my consciousness.” (P4).

Thus, strong feedback loops associated with the tool actually fed faculty with enough information to consistently remind them of the tangibility of their efforts in providing feedback to trainees, which was essential to ensuring the tool’s survivability.

## Discussion

Our qualitative study sought to illuminate how learning culture (LC) might influence the survivability of an online feedback tool in a clinical learning environment based on its perceived usefulness. Through the sociocultural lens of LC, feedback and feedback conversations were viewed as complex, dynamic and bidirectional interpersonal encounters – never merely ‘top down’ exchanges [34].

Within our LC, the tension for clinical teachers was prominent as clinical work often took precedence as compared to teaching duties. This reflected a structural and systemic issue: clinical teachers bore a dual burden of teaching and managing clinical responsibilities, and were expected to be performing in both. This fact intensified faculty’s cognitive load, limiting meaningful engagement not only with online feedback tools but also with the feedback system as a whole. This situation might already predispose faculty to provide less feedback. Hence, institutional support needs to extend beyond acknowledging, reimagining and merely creating on paper a solid support system and environment for faculty. A strong system to develop and support faculty as both clinicians and educators will be prudent [35]. These roles are not ‘either or’, but collective roles that we need to future-proof for all faculty in health professions education. The system should also strongly support measures to legitimize feedback not only for summative purposes but, more importantly, for formative purposes [2]. Institutional culture is the ‘social glue’ of any organisation [36]. However, institutional and learning cultures within healthcare professions education may valorise clinical productivity over educational contributions [37]. An inclination towards gathering reflections of performance through summative feedback over formative feedback may directly or indirectly marginalize tools that support feedback and limit the full potential of feedback [38]. Our study resonates with existing knowledge that, unless there are explicit frameworks within the institution that would evolve to legitimize and reward the use of such feedback tools within the system, their survivability and serviceability will remain limited, regardless of their technical robustness.

Our results also show that using a written feedback (WF) tool presents unique challenges stemming from prevailing beliefs about WF. WF is increasingly recognised as an important, unexplored area of feedback conversation with both untapped potential [39] and hidden challenges. In our study, both experienced and junior faculty undervalued WF due to their limited understanding of its purpose and benefits, resulting in scepticism toward the tool. This result signifies a worrying conceptual gap within the LC that may undermine the feedback tool’s survivability. Undervaluing WF may also explain why some participants used the tool frequently at first but eventually stopped using it altogether. Feedback, to many faculty, simply meant providing comments, whether verbal or written. Faculty were unclear about the roles of verbal and WF. Faculty often perceived WF as a duplicate to verbal feedback. This misconception, coupled with the lack of feedback loops, left many faculty losing the motivation to provide feedback, especially WF, which was already deemed punitive and risky. Feedback delivery, even via a tool, proved to be cognitively and emotionally challenging to faculty. While technological tools could augment interactions and improve feedback system adoption [40,41], we must not assume every faculty member is well equipped with feedback knowledge and skills. Similarly, we cannot assume faculty will readily accept tools deemed effective for the learning environment. As our study shows, there may be no corresponding changes in mindset, values and practices embedded within the LC. Thus, faculty development must address not just faculty’s usage of the tool, but also their values and beliefs about the tool. Cultural readiness matters as much for survivability as technological or innovation readiness.

Our results also illustrate that psychological safety and trust are critical influences on a tool’s uptake and usage. Unaddressed faculty fears of mishandling, judgement, or misinterpretation may disrupt engagement with the tool, that would then affect its survivability within the learning culture. This insight reveals the dangers of implementing a tool without first assessing the readiness on the ground to shift to mindsets to be conducive to feedback. Studies have shown that the introduction of any intervention, including a simple tool, has implications for institutional trust, sparking questions about the role and impact of digital surveillance [42] and faculty autonomy [43]. Hence, without a well-thought strategy to build trust, even the most effective and well-intentioned tools may be perceived as threats. This situation may require transparency on how the data is stored, used and communicated, putting faculty autonomy at the forefront in order to provide a sense of academic freedom [44], while balancing digital surveillance required for such online tools. This academic freedom has to be coupled with strong centralized support with interventions to promote consistent understanding of the usage of the digital tool or innovation to enable widespread and longitudinal survival of the tool. Faculty input should be actively sought, or, even better, involve faculty as beneficiaries of the tool or innovation to enable them to appreciate its value.

Our findings also draw attention to the transient nature of faculty-learner relationships as a common feature of the clinical learning culture that others have observed [45,46]. We found that the transient nature of relationships hindered the continuity, depth and effectiveness of feedback loops. Given the power of the relational aspect of learning culture [47], feedback should be viewed as an interaction, a conversation [13], that requires continuity and enhanced rapport building. Within a feedback system and learning environment structured as episodic encounters, feedback tools must account for such structural challenges to relational conditions. It is crucial that the building and maintenance of such relational aspects not stem only from faculty, but learners as well. Hence, within the learning environment, one of the ways to mitigate this structural challenge is by shifting our focus from summative feedback, as a reflection of good performance and growth, to formative feedback. Deliberate mechanisms need to be built in to mend the cracks created by fleeting relationships in the LC to ensure the feedback loop is closed effectively, in order to ensure continual motivation among faculty to use the tool. Feedback should be taught and viewed as everyday communicational events, utilising the tool to capture important moments of interactions or conversations, where actions and meaning are co-constructed by faculty and learners [48]. Without this in mind, even the most well-designed tools may fail to close the feedback loop, and will not survive.

## Limitations

This study is limited to the perspectives of faculty. Trainees’ perspectives and experiences are also important considerations and should be the focus of future research.

## Significance, Impact and Conclusion

This study informs future strategies of developing written feedback tools, contextualized to the learning culture of a particular setting. Our study suggests that one of the most important factors influencing an online tool’s survivability is not technical in nature. Instead, users’ perceptions and conceptualization of written feedback wields greater influence. Learning culture is the driver that subtly yet deeply impacts the belief system, perspectives and practices of feedback among faculty. We call on educators and program leaders to adopt a careful, proactive and iterative approach to designing any innovation or tool, embracing complexity and socio-cultural concerns from the outset. A tool that truly survives is one that withstands the tests of implementation, adoption, and, not only attains sustainability, but also, more importantly, thrives.

## Previous Presentations

This research was presented at the short communications section in AMEE Conference in August 2024.

## Additional File

The additional file for this article can be found as follows:

10.5334/pme.2166.s1 Appendices. Appendix 1 and 2.
