# Supplementary material for: How Learning Culture Influences the Survivability of an Online Feedback Tool
Source: Perspect Med Educ. 2026 Mar 23;15(1):296–307. doi: 10.5334/pme.2166 (PMC13025158; doi:10.5334/pme.2166)
Supplement: Appendices. — Appendix 1 and 2. [file pme-15-1-2166-s1.zip › pme-2166_khoo-s1/Appendix 1.docx]

(1) A summarised version of participants’ profile and different phases of interviews

| Phase of Interview | Number of Participants | Position | Years of experience | Frequency of feedback platform usage |
| --- | --- | --- | --- | --- |
| Initial phase | 3 | 2 consultants, 1 senior consultant | 12 to 30 years | Frequent users |
| Mid Phase 1 | 2 | 1 Associate Consultant, 1 Senir Consultant | 10 to 20 years | Infrequent users turned non-users |
| Mid phase 2 | 2 | 1 Associate Consultant, 1 Senior Consultant | 10 to 25 years | Initial adopters turned non-users |
| Final Phase | 3 | 1 Associate Consultant, 1 Senior Consultant | 10 to 30 years | Combination of initial non-users or infrequent users, turned late adopters |

(2) Detailed version of sequence of participant sampling and their profile
