# Supplementary material for: How Learning Culture Influences the Survivability of an Online Feedback Tool
Source: Perspect Med Educ. 2026 Mar 23;15(1):296–307. doi: 10.5334/pme.2166 (PMC13025158; doi:10.5334/pme.2166)
Supplement: Appendices. — Appendix 1 and 2. [file pme-15-1-2166-s1.zip › pme-2166_khoo-s1/Appendix 1.pdf]

(1) A summarised version of participants' profile and different phases of interviews

Phase of Interview	Number of Participants	Position	Years of experience	Frequency of feedback platform usage
Initial phase	3	2 consultants, 1 senior consultant	12 to 30 years	Frequent users
Mid Phase 1	2	1 Associate Consultant, 1 Senir Consultant	10 to 20 years	Infrequent users turned non-users
Mid phase 2	2	1 Associate Consultant, 1 Senior Consultant	10 to 25 years	Initial adopters turned non-users
Final Phase	3	1 Associate Consultant, 1 Senior Consultant	10 to 30 years	Combination of initial non-users or infrequent users, turned late adopters

(2) Detailed version of sequence of participant sampling and their profile

Participant	Faculty Position	Years of experience (as Faculty/ Supervisor)	Frequency of feedback platform usage
P1	Senior consultant	30	Frequent user
P2	Consultant	12	Frequent user
P3	Consultant	18	Frequent user
P4	Senior Consultant	20	Initial adopter, then non-user
P5	Associate Consultant	10	Infrequent, then non-user
P6	Senior Consultant	25	Infrequent, then non-user
P7	Associate Consultant	10	Initial adopter, then non-user
P8	Associate Consultant	10	Initial infrequent user, then used more frequently later
P9	Senior Consultant	25	Initial non-user, then late adopter
P10	Senior Consultant	30	Initial non-user, then late adopter
