# Supplementary material for: How Learning Culture Influences the Survivability of an Online Feedback Tool
Source: Perspect Med Educ. 2026 Mar 23;15(1):296–307. doi: 10.5334/pme.2166 (PMC13025158; doi:10.5334/pme.2166)
Supplement: Appendices. — Appendix 1 and 2. [file pme-15-1-2166-s1.zip › pme-2166_khoo-s1/Appendix 2.pdf]

(1) How the online feedback platform works

KKH Children's Emergency
Faculty Feedback Form
2nd Version

1.

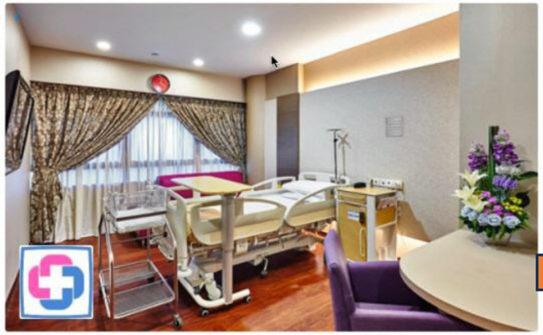

The first view that supervisors see when they access the form digitally from desktop, phones or tablet

Supervisors are then prompted to select the setting in which feedback/ encounter/ observation took place

5.

*What is the setting you are proving feedback for?

Single choice

- Clinical / during shift
- Educational activity
- Research/ scholarly activity
- Administrative work
- Written feedback from parent/ patient
- Others

6.

*Which of the following areas would you like to provide feedback on?

Supervisors then chose from any of these 6 ACGME criteria they would like to provide feedback on (they can choose more than one option if applicable)

- (A) Medical Knowledge: basic sciences

  e.g. basic sciences, clinical knowledge
- (B) Systems based practice

  e.g. identify errors and helps with solution, Advocate for quality care
- (C) Professionalism

  e.g. Self awareness of limitations; help seeking behaviour; Flexibility and Maturity; Leadership; Self Confidence; Punctuality; Appearance and Care; Teamwork and responsibility
- (D) Practice-based learning and improvement

  e.g. Use of electronic health records; Motivation for self- Education; Teaching others
- (E) Patient care

  e.g. History and Examination; Organisation and Prioritisation of Responsibilities; Clinical decision making (diagnostic and therapeutic); Disposition Plan (including patient education and appropriate referrals); Procedures (including indications, technical skills and choice of sedation/ pain relief); Emergency Stabilisation
- (F) Interpersonal skills and communication

Supervisors then type out the details of the encounter/ observation/ discussion followed by the feedback

(2) <u>An example of how the free-text feedback submitted by the supervisor (s) looked like</u>

Very conscientious and responsible in reading up on topics around cases and has a good base of medical knowledge. Saw a case triaged as pertussis and a case of infantile pyrexia in a GBS positive mother. Told him that he should read up around the topics. A week later when I went up to quiz him, he was able to describe the course of illness in pertussis early, understand complications and other associated features like lymphocytosis. For the GBS case, he was able to clearly define both early and late onset GBS sepsis. This positive and self driven learning attitude will help boost his bank of medical knowledge which will help with patient care.
There was a little bit of a lull in between patients and [redacted] went up to introduce himself to the new MOs who joined the team. After some brief introduction and small talk he took them on a mini tour round the main CE area sharing the tips and tricks of ordering meds, tracing results and practical daily insights into work. The MOs were pleased and asked many important questions that helped improve their knowledge of CE's workflows. It's nice to see how [redacted] effortlessly slipped into this role of mentor sharing his personal knowledge with new trainees to help them assimilate. Shows good initiative and leadership qualities.
You have consistently demonstrated a strong sense of responsibility and team spirit in your willingness to do more than your expected duties within our team (modular team system during COVID-19 crisis). Examples include these: (1) When you were rostered for a 2 hour later start one morning shift (I had deliberately planned this to balance out work hours among our doctors), you checked with me why you shouldn't come 2 hours earlier, instead of just accepting the benefit. (2) Volunteering to cover isolation duties (seeing suspected COVID-19 patients) more often as needed, without comparing the contributions of colleagues / whose turn it is. (3) Volunteering to let colleagues take their breaks first before you take your own. (4) 15 minutes from the end of a 12 hour shift, you proactively went to see a P3 patient who had just arrived in isolation area, when it would have been reasonable to leave it to the incoming team to see this very stable patient who had just arrived.

The platform captures written feedback from various shift supervisors, which are then consolidated into an excel file by the administrative team. Specific feedback collated for each trainee are then delivered to the posting supervisor who would then utilise the feedback as discussion points during the supervisor-supervisee meeting. The provider of the feedback is anonymised.

(3) <u>The monitoring of the online feedback platform: usage and categories submitted</u>

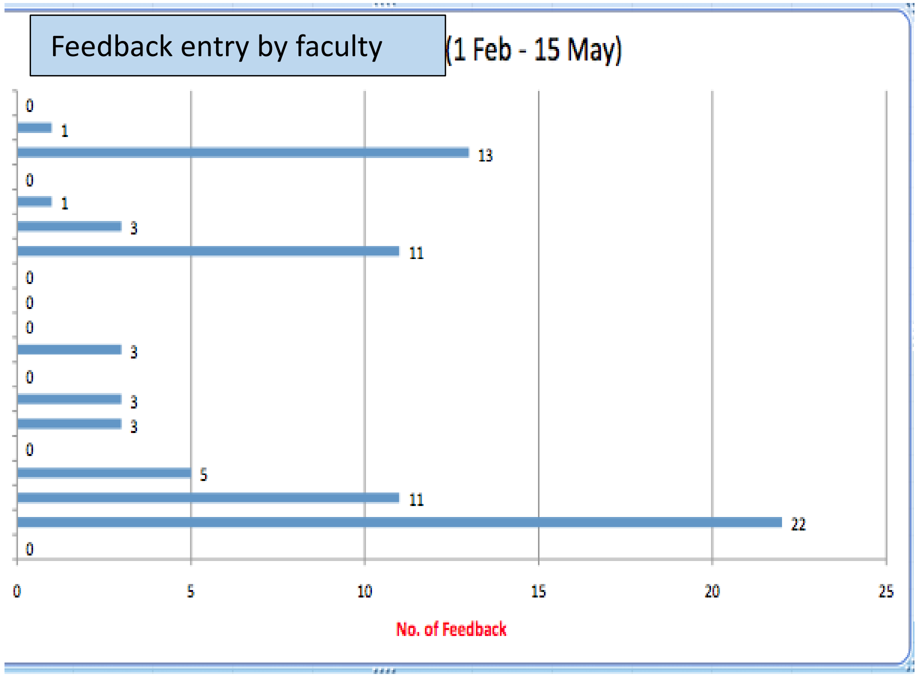

Names of the supervisors appear on the left column (removed from this picture). These are only visible to SA and AT for monitoring and identifying faculty who may need support and encouragement to provide feedback.

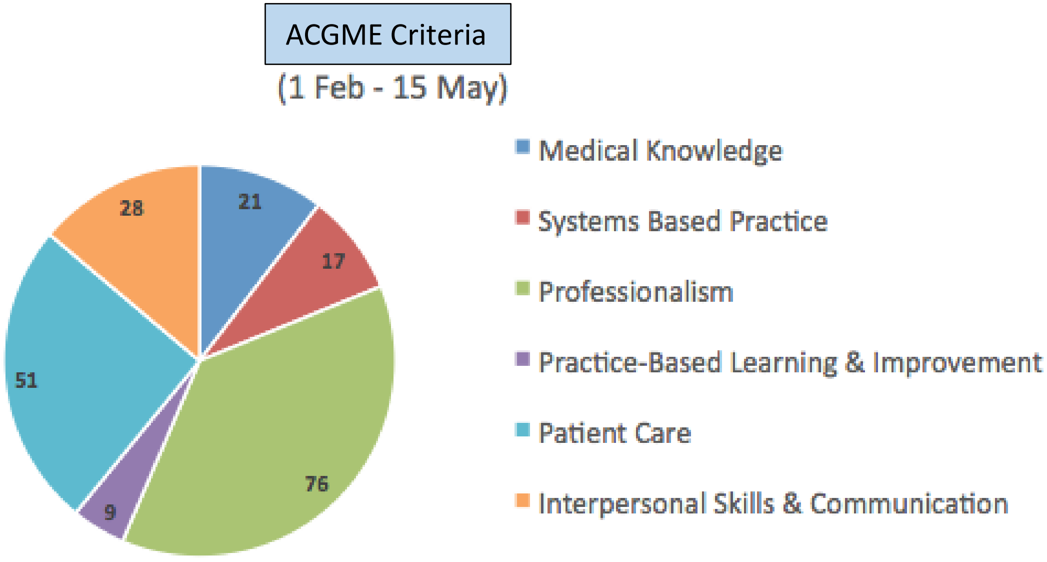

Monitoring the submissions of feedback based on ACGME categories- to encourage supervisors to observe and provide feedback holistically on all areas as supervisors initially were mostly focused on medical knowledge and patient care.
